# Psychometric study of the Maslach Burnout Inventory-Student Survey on Thai university students

**DOI:** 10.1038/s41598-024-84829-8

**Published:** 2025-01-13

**Authors:** Ernesto López-Gómez, Raúl González-Fernández, Buratin Khampirat

**Affiliations:** 1 Universidad Nacional de Educación a Distancia (UNED), 28045 Marid, Spain; 2 Institute of Social Technology, Suranaree University of Technology, Nakhon Ratchasima, 30000 Thailand

**Keywords:** Maslach burnout inventory, University students, Scale validation, Self-report, Confirmatory factor analysis, Psychometric properties, Psychology, Risk factors

## Abstract

The Maslach Burnout Inventory-Student Survey (MBI-SS) is a widely used instrument to assess burnout levels, which provides valuable insight into their psychological well-being. Accurate measurement of burnout is crucial for developing interventions aimed at reducing stress and promoting mental health among students. This study aims to validate the MBI-SS when applied among Thai university students and to examine whether the psychometric properties of the scale are consistent with the original conceptual framework. A total of 413 undergraduate students from Thailand participated in the study, with 57.63% females and 42.37% males, and a mean age 21.75 years (*SD* = 2.40). The MBI-SS was translated into Thai by following rigorous procedures to maintain accuracy and cultural relevance. The factorial structure of the MBI-SS Thai version was evaluated using confirmatory factor analysis (CFA) for both a three-factor model and second-order factor model. The Thai version of the MBI-SS demonstrated a three-dimensional structure consistent with the original inventory, with excellent model fit indices. All item factor loadings exceeded the recommended threshold, and the instrument showed high internal consistency, establishing it a valuable tool for future research and practical application in educational settings aimed at addressing and reducing student burnout.

## Introduction

Mental health is one of the primary determinants of quality of life, well-being, social, and work satisfaction^1,2^. However, concerns about university students’ mental health and well-being have been increasingly by higher education institutions worldwide^3^. Evidence suggests that mental health stress of students often begins when they enter university and escalates in both frequency and severity over time^4,5^. This growing stress and burnout not only affected academic performance but also their overall life and future career^6^. Additionally, students are at risk of developing general mental disorders such as panic disorder, depression, anxiety, and social phobia, with many of these conditions first manifesting in childhood or adolescence^7^. The prevalence of these disorders tends to increase throughout adolescence and peaks in early adulthood, around the age of 25^7–9^, a period during which university undergraduates are particularly vulnerable compared to the general population in both developed and developing countries^10^. Furthermore, research by Dyrbye et al.^11^ highlighted that burnout rooted in academic stress accelerates the onset of work-related burnout later in life.

Although there has been a growing global interest in investigating and addressing students’ mental health problems, there has also been a rise in the prevalence of depression, anxiety, stress, self-harm, and suicidal ideation among university students. Examples of these studies were conducted in various countries such as Bangladesh^12^, China^13^, Ethiopia^14^, Malaysia^15^, Poland^16^, Saudi Arabia^17^, South Africa^18^, South Korean^19^, Sudan^20^, the United States, and Canada^18,21^. The consistency of findings across countries highlights the global nature of this problem, which urgently needs to be addressed. Research further indicates that university admission can be a stressful and overwhelming experience for some students^22,23^. This stress is often compounded by high academic standards^14^, imbalances between academic commitments and personal life^24^, and the financial pressures^25^. These factors contribute to an increased risk of other mental health issues and future career burnout^26,27^. Previous studies have shown that academic burnout is linked to the university environment, faculty, support staff, assignments and workload (e.g., submission of assignments and preparation for presentations and exams)^28^, and perfectionism^19^. Burnout is exacerbated when students are unable to meet expectations or perceive a gap between their personal resources (both internal and external)^29^. This notion is related to beliefs in self-efficacy^30^, self-esteem and professional self-efficacy^31^, suicidal ideation^32^, physical and mental stress^33^, poor academic performance, and dropout intention^34^. At the university level, the combination of high cognitive demands and time pressure often leads to chronic stress, which can ultimately result in academic burnout. Given the significant relationship between burnout and these adverse outcomes, it is relevant to have a reliable tool to measure burnout in an academic context^34^. Burnout is not simply characterized by exhaustion or an overwhelming workload; individuals with burnout also experience a loss of connection to their academic tasks, which impacts both their motivation and identity^35^. Furthermore, Lin and Huang^36^ hypothesized that student burnout may adversely affect the relationships of students with their university, peers, and the academic staff.

In the past few years, the academic burnout of university students has been assessed using a modified version of the Maslach Burnout Inventory-General Survey (MBI-GS) of Schaufeli et al.^37^, which was designed for application to occupation groups apart from service occupations and education sectors. This tool (MBI-GS) was later adapted to evaluate burnout among college and university students with Schaufeli et al.^38^, using the Burnout Inventory-Student Survey (MBI-SS) to assess its factorial validity, construct validity, and measurement invariance in students population from Spain, Portugal, and the Netherlands. The results of confirmation factor analysis (CFA) demonstrated that the MBI-SS measurement structure, with its three expected components of the modified version of the MBI-GS (i.e., exhaustion, cynicism, and professional efficiency), demonstrated a good fit to the data. Schaufeli et al.^38^ defined student academic burnout as a combination of the feeling of exhaustion due to educational demands, a cynical and indifferent attitude to education, and a feeling of incompetence in education, including reduced academic efficacy as a learner.

As a tool for assessing stress levels, MBI-SS has been the subject of much research in university students globally and has been extensively validated in various contexts^39–44^. In Thailand, Puranitee et al.^45^ and Wongtrakul et al.^46^ conducted a study on the psychometric properties of the MBI-SS among undergraduate medical students at one university. These studies illustrated that the MBI-SS possessed good reliability and validity. However, a significant limitation of the study on MBI-SS validation in Thailand is the lack of diversity of the populations. Most studies have focused exclusively on medical students from a single institution, which may not be representative of the broader Thai student population. Moreover, further research is needed to evaluate the psychometric properties of the MBI-SS across languages and cultural contexts. Given the cultural differences between Thailand and other countries, understanding the extent to which the findings of previous studies on the MBI-SS are applicable to the Thai context is important. The reason is that doing so will help to ensure that the MBI-SS is appropriately and effectively used in Thailand. To address this limitation, future research should aim to include a more diverse range of individuals, including those from different cultural and educational backgrounds as well as age groups and gender.

Previous research has demonstrated the adequate measurement validity and reliability of the MBI-SS across various contexts. However, the application of MBI-SS to various countries may need to undergo minor modifications in some items to enhance the suitability of the measurements^41,47–49^. One can infer that even if the validity and reliability of the MBI-SS can be demonstrated, confirming its measurement properties is still required before using it can be used on different samples, because adjustments are occasionally required to render the scale more appropriate. Especially for undergraduate students in Thailand, where the academic and societal context may differ from that of other countries.

Having a validated instrument that can early identify students at risk for burnout will be an opportunity for preventive strategies^50^. Information obtained in addition can be useful for further research and provide valuable insights for practitioners and policymakers. Therefore, this study aims to validate the MBI-SS when applied among Thai university students and to examine whether the psychometric properties of the scale are consistent with the original conceptual framework, which would ensure that the MBI-SS retains its theoretical underpinnings and cultural relevance. This study thus contributes to addressing that gap by adapting and validating the MBI-SS for a broader range of Thai undergraduate students, making it one of the first studies to investigate the scale’s applicability across multiple fields of study in Thailand. This research also provides recommendations for future studies examining the impact of cultural diversity and educational environments on burnout.

## Methods

The participants consisted of 413 undergraduate students from 34 universities in Thailand (238 women [57.63%], 175 men [42.37%]). The mean age was 21.75 years (*SD* = 2.40). In terms of year level, 20.34% (n = 84), 9.20% (n = 38), 25.91% (n = 107), 38.26% (n = 158), and 6.30% (n = 26) were in the first to fifth years and higher, respectively. In terms of residence, 56.90% (n = 235) of participants live in urban areas, while 43.10% (n = 178) reside in rural zones. Further demographic details are provided in Table 1.

**Table 1:** Demographic characteristics of the participants by gender.

Demographic		Men		Women		Total	
		n	%	n	%	n	%
Age	< 20 year	30	17.14	38	15.97	68	16.46
	20–25 year	139	79.43	197	82.77	336	81.36
	> 25 year	6	3.43	3	1.26	9	2.18
	Total	175	100.00	238	100.00	413	100.00
Year of study	First	37	21.14	47	19.75	84	20.34
	Second	11	6.29	27	11.34	38	9.20
	Third	33	18.86	74	31.09	107	25.91
	Fourth	83	47.43	75	31.51	158	38.26
	Fifth and higher	11	6.29	15	6.30	26	6.30
	Total	175	100.00	238	100.00	413	100.00
GPA (scale ranges from 0.00 to 4.00)	< 2.00	8	4.68	3	1.30	11	2.74
	2.00–2.50	33	19.30	45	19.57	78	19.45
	2.51–3.00	23	13.45	42	18.26	65	16.21
	3.01–3.50	65	38.01	92	40.00	157	39.15
	> 3.50	42	24.56	48	20.87	90	22.44
	Total	171	100.00	230	100.00	401	100.00
Father’s highest educational level	Did not attend school	1	0.57	1	0.42	2	0.48
	Elementary school	17	9.71	17	7.14	34	8.23
	Lower-secondary	5	2.86	12	5.04	17	4.12
	Upper-secondary	12	6.86	23	9.66	35	8.47
	High vocational diploma	18	10.29	39	16.39	57	13.80
	Bachelor’s degree	92	52.57	101	42.44	193	46.73
	Master’s degree	20	11.43	27	11.34	47	11.38
	Doctorate degree	7	4.00	11	4.62	18	4.36
	N/A	3	1.71	7	2.94	10	2.42
	Total	175	100.00	238	100.00	413	100.00
Mother’s highest educational level	Did not attend school	2	1.14	1	0.42	3	0.73
	Elementary school	18	10.29	29	12.18	47	11.38
	Lower-secondary	9	5.14	12	5.04	21	5.08
	Upper-secondary	12	6.86	16	6.72	28	6.78
	High vocational diploma	18	10.29	37	15.55	55	13.32
	Bachelor’s degree	85	48.57	98	41.18	183	44.31
	Master’s degree	25	14.29	27	11.34	52	12.59
	Doctorate degree	4	2.29	14	5.88	18	4.36
	N/A	2	1.14	4	1.68	6	1.45
	Total	175	100.00	238	100.00	413	100.00
Residential zone	Rural	78	44.57	100	42.02	178	43.10
	Urban	97	55.43	138	57.98	235	56.90
	Total	175	100.00	238	100.00	413	100.00

### Instruments

The questionnaire consisted of two parts. Part A collected sociodemographic information (e.g., gender, age, and year of study), while Part B comprised the MBI-SS, developed by Schaufeli et al.^38^ to assess student burnout in terms of academics. A license for the use of MBI questionnaire was sought and granted by Mind Garden, Inc. The MBI-SS contains 15 items that assess three dimensions, namely, emotional exhaustion (EX, five items), cynicism (CY, six items), and professional efficiency (PE, four items). Items were rated using a seven-point Likert scale ranging from 0 (never) to 6 (always). Each dimension was calculated by averaging the item scores. High scores on the Emotional Exhaustion and Cynicism dimensions, combined with low scores on Professional Efficacy, indicate the presence of academic burnout, as the Professional Efficacy items are presented using positive words.

The MBI-SS was modified to be the Thai version by using translation and cultural adaptation. The first step in preparing the questionnaire involved translating it from English to Thai by two Thai-speaking researchers. A discussion followed to address any similarities or discrepancies in the translations. To ensure conceptual equivalence with the original instrument and the meaning was preserved accurately, two specialists in English-Thai translation reviewed and back-translated the questionnaire. Subsequently, a pilot testing of the Thai version was conducted with a sample of 20 Thai students to examine question clarity and adjust culturally inappropriate terminology to ensure that the instrument was relevant to the cultural and educational context of Thai students. These modifications addressed potential differences in the interpretation of burnout-related constructs, while maintaining the core structure and validity of the original MBI-SS.

### Procedure

The study collected data using multiple methods, including in-person gathering in conventional classrooms and conference rooms, as well as through online platforms. To ensure that this research is transparent and complies with ethical standards for human research, the researchers informed the participants of the objective of the study and assured them of the confidentiality of their information. All responses were anonymous and presented as an overview, in aggregate form, without individual identifiers. Furthermore, participants were free to decline participation or withdraw at any point if they felt uncomfortable. On average, completing the questionnaire took approximately 15–20 min.

### Data analysis

Data analyses were conducted using SPSS 29.0 and Mplus 8.3. The initial step in the data analysis involved organizing the data obtained from the questionnaire, which included checking for errors, handling missing values, and ensuring that the dataset was appropriately formatted for analysis. Descriptive analysis was then performed to calculate the basic statistics such as mean, standard deviation, skewness, and kurtosis. This step provided a summary of the data and helped identify outliers or skewness^51^. Following the descriptive analysis, the validation of the scale to assess the extent to which the MBI-SS measures the construct it was designed to measure. This process involved correlation and construct validity analyses, which included both exploratory factor analysis (EFA) and confirmatory factor analysis (CFA).

The main purpose of EFA is to explore and determine the structure of a scale by reducing the number of existing items and constructing a new small set of items to obtain a new factor structure that best fits that small set of items. CFA was conducted to ensure that the adapted Thai version of the MBI-SS maintains its construct validity within the Thai student population. In the CFA, several fit indices were employed to assess how well the model fits to the data. The commonly used fit indices are chi-square statistic and its associated degrees of freedom (χ^2^/*df*), comparative fit index (CFI), the Tucker–Lewis index (TLI), standardized root mean square residual (SRMR), and root mean square error of approximation (RMSEA). These indices impose specific criteria for determining model fit, such as *χ*^2^*/df* (acceptable if < 3), CFI and TLI values ≥ 0.95, SRMR and RMSEA values ≤ 0.06 and 0.08 respectively. To compare the models, the Akaike information criterion (AIC) was used, with lower AIC values indicating a better fit. Finally, the homogeneity of the scales was examined through standardized factor weights (values greater than 0.40 are typically regarded acceptable)^52,53^. Meanwhile, scale reliability was assessed using Cronbach’s alpha, McDonald’s omega, composite reliability (CR), and the average variance extracted (AVE). Cronbach’s alpha was calculated to indicate the internal consistency reliability of the scale under the assumption that multiple items measure the same underlying construct. Where Cronbach’s alpha ≥ 0.70 was considered satisfactory. McDonald’s Omega is an alternative reliability index to measure internal consistency of factors. The value of Omega is 0.70 or higher, indicating the internal consistency of the factors is acceptable. CR and AVE were used to assess the convergent validity of the measurement model. A CR value of 0.60 or above indicates a set of indicators or items underlying the same concept. While AVE should be greater than 0.50, which indicates that the construct captures more variance in the items than measurement error.

### Ethical statements

Informed consent was obtained from all participants involved in the study. The research was conducted in strict adherence to ethical standards, following the principles outlined in the Declaration of Helsinki, and was approved by the Human Ethics Committee of Suranaree University of Technology (approval number: EC-63-85).

## Results

### Descriptive statistics

The descriptive analysis of the MBI-SS items, as shown in Table 2, demonstrated that item EX5 (“I feel burned out from my studies”) obtained the highest mean score (*M* = 4.25, *SD* = 1.312). In contrast, PE2 (“I believe that I make an effective contribution to the classes that I attend”) exhibited the lowest mean score (*M* = 2.65, *SD* = 1.62). The skewness ranged from − 0.687 to 0.302, while Kurtosis ranged from − 1.063 to 0.440, indicating that the response distribution for each item was roughly normal. To evaluate the item-total correlations, the Corrected Item Total Correlation (CITC) was used, demonstrating that all items exceeded the acceptance criterion of 0.30, with CITC values ranging from 0.809 to 0.954. This confirm that each item significantly contributed to the overall consistency of the scale.

**Table 2:** Items, descriptive statistics, and results of exploratory factor analysis (EFA) for MBI-SS (with varimax rotation).

Items		Descriptive statistics						Results of EFA		
		*M*	*SD*	*SK*	*KU*	*CITC*	Dimensions	1	2	3
EX1	Emotionally drained	4.00	1.225	− 0.374	− 0.103	0.819	Emotional exhaustion	–	0.841	–
EX2	Used up	3.75	1.336	− 0.687	0.440	0.813		–	0.744	–
EX3	Tried in the morning	4.03	1.370	− 0.440	− 0.301	0.825		–	0.807	–
EX4	Classes all day	3.98	1.389	− 0.585	− 0.001	0.833		–	0.827	–
EX5	Burned out	4.25	1.312	− 0.540	− 0.053	0.809		–	0.817	–
CY1	Less interested	3.95	1.329	− 0.237	− 0.542	0.872	Cynicism	–	–	0.803
CY2	Less enthusiastic	3.88	1.295	− 0.319	− 0.229	0.857		–	–	0.785
CY3	Cynical	3.85	1.412	− 0.469	− 0.010	0.867		–	–	0.827
CY4	Doubt significance	3.93	1.356	− 0.507	0.063	0.822		–	–	0.801
PE1	Solve problems	2.82	1.706	0.300	− 0.967	0.911	Professional efficacy	0.932	–	–
PE2	Effective contribution	2.65	1.620	0.142	− 0.993	0.929		0.946	–	–
PE3	Good student	2.86	1.801	0.201	− 1.063	0.917		0.937	–	–
PE4	Exhilarated	2.67	1.774	0.230	− 1.047	0.933		0.948	–	–
PE5	Accomplished	2.69	1.769	0.302	− 0.971	0.943		0.956	–	–
PE6	Confident	2.71	1.798	0.255	− 1.019	0.954		0.966	–	–
KMO								0.931		
Bartlett’s test of sphericity								Chi-Square = 7693.950, *p* < 0.001		
Eigenvalues								7.363	4.590	0.807
Percent of total variance explained								49.085	30.602	5.381
Cumulative percent								49.085	79.687	85.068

Note. SE of SK = 0.119, SE of KU = 0.238.

### Item response analysis

Table 3 presents the distribution of responses for each MBI-SS item based on a scale from 0 (never) to 6 (always). The prevalence rates varied from 0.24% to 31.23%. EX2 obtained the highest prevalence rate (rating of “often”).

**Table 3:** Item response analysis.

Items		Never	Almost never	Occasionally	Sometimes	Often	Almost always	Always
EX1	n	1	14	20	110	114	112	42
	%	0.24	3.39	4.84	26.63	27.60	27.12	10.17
EX2	n	12	15	32	98	129	101	26
	%	2.91	3.63	7.75	23.73	31.23	24.46	6.30
EX3	n	2	22	24	96	104	103	62
	%	0.48	5.33	5.81	23.24	25.18	24.94	15.01
EX4	n	5	22	25	83	118	105	55
	%	1.21	5.33	6.05	20.10	28.57	25.42	13.32
EX5	n	1	17	14	80	116	103	82
	%	0.24	4.12	3.39	19.37	28.09	24.94	19.85
CY1	n	2	12	41	108	95	100	55
	%	0.48	2.91	9.93	26.15	23.00	24.21	13.32
CY2	n	2	18	29	112	111	97	44
	%	0.48	4.36	7.02	27.12	26.88	23.49	10.65
CY3	n	9	19	30	102	113	88	52
	%	2.18	4.60	7.26	24.70	27.36	21.31	12.59
CY4	n	7	14	34	90	118	101	49
	%	1.69	3.39	8.23	21.79	28.57	24.46	11.86
PE1	n	20	103	66	91	45	55	33
	%	4.84	24.94	15.98	22.03	10.90	13.32	7.99
PE2	n	33	98	57	98	58	58	11
	%	7.99	23.73	13.80	23.73	14.04	14.04	2.66
PE3	n	32	95	56	85	48	57	40
	%	7.75	23.00	13.56	20.58	11.62	13.80	9.69
PE4	n	43	99	57	80	51	57	26
	%	10.41	23.97	13.80	19.37	12.35	13.80	6.30
PE5	n	37	102	61	85	44	52	32
	%	8.96	24.70	14.77	20.58	10.65	12.59	7.75
PE6	n	42	96	59	83	46	54	33
	%	10.17	23.24	14.29	20.10	11.14	13.08	7.99

### Exploratory factor analysis

Table 2 presents the EFA results with varimax rotation, which were used to investigate the grouping of items into factors and assess whether the data support the proposed model. The results of the Kaiser–Meyer–Olkin (KMO) test and the Bartlett’s sphericity test indicated that the data were suitable for EFA. The KMO value was very high at 0.931, suggesting that a significant proportion of variance in the variables may be attributed to common factors. Bartlett’s sphericity test was also significant (chi-square = 7693.950, *df* = 105, *p* < 0.001), which confirms that the variables were sufficiently related and suitable for testing the structure using EFA. Moreover, EFA revealed that the MBI-SS consists of a three-factor solution, which accounts for 85.068% of the total variance. Factor 1 corresponded to the Professional Efficacy dimension (PE1–PE6) with loadings ranging from 0.932 to 0.966. Factor 2 represented the Emotional Exhaustion dimension (EX1–EX5), with loadings ranging from 0.744 to 0.841. Finally, Factor 3 was associated with the Cynicism dimension (CY1–CY4), with loadings ranging from 0.785 to 0.827.

### Factorial validity

**Fig. 1:**
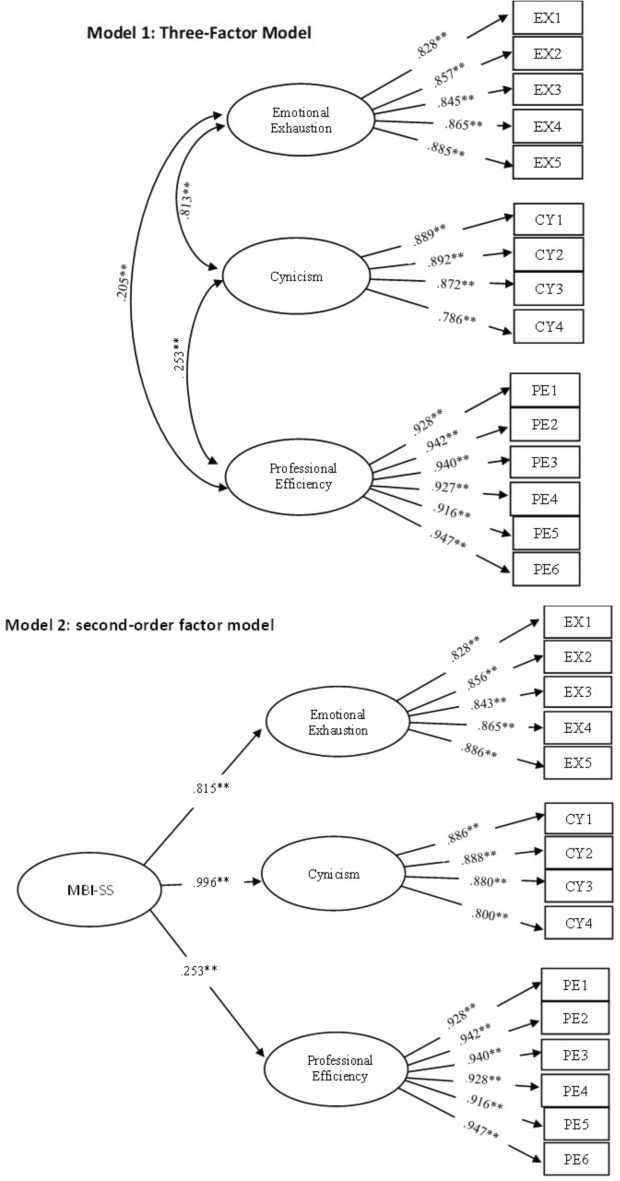
CFA Results with standardized factor loadings of the three-factor model (Model 1) and second-order factor model (Model 2).

The study tested for the factorial validity of the MBI-SS using CFA (Fig. 1). The CFA results demonstrated a good fit of the MBI-SS in both models, namely, the three-factor model (Model 1: χ^2^ = 169.873, *df* = 70, *p* < 0.001; χ^2^/*df* = 2.427, CFI = 0.981; TLI = 0.973; RMSEA = 0.062 [90% CI: 0.050 to–0.074]; SRMR = 0.028) and the second-order factor model (Model 2: χ^2^ = 192.013, *df* = 72, *p* < 0.001; χ^2^/*df* = 2.667, CFI = 0.984; TLI = 0.977; RMSEA = 0.067 [90% CI: 0.056 to–0.078]; SRMR = 0.029). The standardized factor loadings of the items (λ) of both models ranged from 0.786 to 0.947, which exceeded the recommended threshold (> 0.40), indicating that each item made a significant contribution to the measured construct. Given the similar model fit and AIC values (AIC: Model 1 = 13,851.561, Model 2 = 13,869.702), both models can be considered appropriate for application among Thai students. However, the composite reliability (CR) and average variance extracted (AVE) values in Table 3 are the values calculated from Model 1.

### Convergent validity

The concept of convergent validity refers to the extent to which a scale (e.g., professional efficacy) correlates with other instruments that assess similar theoretical or conceptual constructs. A higher bivariate correlation between measures of the same construct provides stronger evidence of convergent validity. Table 4 presents the convergent validity evidence for the Emotional Exhaustion (EX) and Cynicism (CY) dimensions, which show significant correlations with measures of mental difficulties. In contrast, the correlations between Professional Efficacy (PE) and mental difficulties are weak, suggesting limited convergent validity for this dimension in relation to mental health outcomes.

**Table 4:** Convergent validity and reliability (Cronbach’s Alpha, McDonald’s Omega, CR, and AVE).

Convergent validity and reliability	Emotional exhaustion	Cynicism	Professional efficacy
Convergent validity (Pearson’s correlation)			
Mental difficulties	0.540**	0.535**	0.153**
Scale reliability			
Cronbach’s Alpha (α > 0.70)	0.930	0.936	0.980
McDonald’s Omega (ω > 0.70)	0.919	0.901	0.968
Composite reliability (CR > 0.6; Model 1)	0.932	0.920	0.976
Average variance extracted (AVE > 0.5; Model 1)	0.733	0.741	0.871

*p ≤ 0.05; **p ≤ 0.001.

### Scale reliability

Internal consistency reliability was used to measure the consistency of items in the same construct. This was assessed using Cronbach’s alpha, McDonald’s omega, composite reliability (CR), and average variance extracted (AVE), as shown in Table 4. Both α and ω were greater than 0.70, indicating a high level of internal consistency. This result implies that the items within each scale are positively correlated with one another and measure the same underlying construct in a consistent manner. Similarly, CR values confirm the high reliability of the latent construct, further supporting strong internal consistency among the items. Additionally, the AVE values were all well above the 0.50 threshold, demonstrating that the constructs explain a significant portion of the variance in the observed variables.

## Discussions

Addressing and decreasing academic and educational burnout require the use of appropriate and validated instruments for measuring psychiatric and psychosocial interventions. Therefore, this study validated the translation of the MBI-SS from English into the Thai version, consisting of three dimensions and 15 items. The psychometric properties of the scale were satisfactory. The EFA results highlighted that the Thai version of the MBI-SS is consistent with previous studies and shows a strong psychometric structure as demonstrated by CFA, which indicates a good model fit^39,42,54^. Furthermore, the measurement models tested included both a three-factor model and a second-order factor model, showing very good fit measures. The three-factor model aligns with the findings of Portoghese et al.^39^, who conducted a study for students in Italy, Aguayo-Estremera et al.^54^ in Spain, and Ilic et al.^55^ in Serbia. However, the current results contrast with the study by Bria et al.^56^ in Romania, where the three-factor model of burnout demonstrated validity only after the omission of one item from the Cynicism dimension. It also includes the test of Turhan et al.^57^ in Germany demonstrated validity for the MBI-SS after removing the professional performance variable.

In the second-order model, the factor loadings of the higher-order construct, MBI-SS, across its three dimensions indicated that Cynicism had the highest loading (0.996), followed by Emotional Exhaustion (0.815) and Professional Efficacy (0.253). This suggests that Cynicism is the most influential dimension that contributes to the overall MBI-SS construct within the context of this model, as it plays a central role in reflecting the emotional boredom and negative attitude of students toward their studies^58,59^.

The results of the three-factor model and the second-order factor model analyses also revealed that the indicators with the highest factor loadings were the same, that is, EX5 (“I feel burned out from my studies”) for Emotional Exhaustion, CY2 (“I have become less enthusiastic about my studies”) for Cynicism, and PE6 (“During class, I feel confident that I am effective in getting things done”) for Professional Efficacy, which indicates a high consistency in the measurement of educational burnout across modeling approaches. Furthermore, this result implies that these indicators are the core of and central to each dimension of burnout among students, indicating that the Thai version of the MBI-SS can reliably measure the underlying construct of burnout, Maslach’s burnout framework.

Evidence of convergent validity was demonstrated by the strong positive correlations between Emotional Exhaustion and Cynicism dimensions with mental health difficulties. This is consistent with the idea that both dimensions reflect disengagement from learning, which is associated with psychological distress^60^. Internal consistency reliability estimates using α, ω, CR, and AVE were as high as in other language versions. Which α values revealed that all three dimensions of the MBI-SS demonstrated internal reliability, meeting the established criterion (α > 0.70), that is consistent with prior research across different countries^40,43,54^. This information further confirms the reliability and stability of the MBI-SS in the Thai student population. However, some studies in other countries have reported lower Cronbach’s alpha coefficients for the scale, falling below the 0.70 threshold^49^. Thus, future research should re-examine the reliability and validity of the MBI-SS before applying it in diverse cultural and contextual settings.

### Strengths and limitations of the study

This study has several strengths. First, the use of a cross-sectional design enabled a comprehensive and multidimensional examination of the Thai version of the MBI-SS, including CFA, to ensure the reliability and validity of the instrument in a new cultural context. Second, the sample size was sufficiently large to provide robust statistical power and reliable results.

However, the study also presents certain limitations. First, due to its cross-sectional nature, the study does not establish a cause-and-effect relationship between burnout and other variables. Second, while the sample size was relatively large, it was limited to university students in Thailand, which may restrict the generalizability of the findings to other populations. Third, the reliance on self-report measures may introduce potential bias, because the participants may over- or under-report their burnout levels due to social desirability, lack of self-awareness, or other subjective factors.

### Implications of the findings

This study confirms acceptable psychometric properties of the Thai version of the MBI-SS, supporting the validity of its factor structure and demonstrating its reliability in assessing burnout risk among university students in Thailand. Moreover, the findings suggest that Thai students may experience burnout differently from students in other countries. Specifically, the second-order CFA results highlight Cynicism and Emotional Exhaustion as the most prominent dimensions of academic burnout in this population. Therefore, to effectively prevent or mitigate burnout, interventions should be tailored to these key dimensions. Indeed, the MBI-SS not only serves as a diagnostic tool but also plays a crucial role in guiding intervention strategies. For instance, universities could implement programs focused on enhancing emotional regulation, improving stress management, and fostering greater engagement with academic work. These insights lay the groundwork for creating comprehensive support systems that not only reduce burnout but also foster students’ long-term academic achievement and personal development^50^.

### Future directions for research

Future research should adopt a longitudinal design to examine the development and progression of burnout over time, providing deeper insights into causal relationships. Understanding the temporal dynamics of burnout could help identify critical periods for intervention and prevention. Additionally, comparative studies involving university students from other countries are recommended to evaluate the universal applicability of the MBI-SS to uncover potential cultural variations in burnout experiences. Such cross-cultural investigations could determine whether the core dimensions of burnout are perceived consistently across different contexts or whether they manifest in culturally specific ways. Multi-group analysis of the burnout measurement model between Thai students and students in other countries should also be tested. Because Thai universities differ from other institutions in many ways, including cultural values, social structures, and academic expectations, all of which affect student life and learning experiences. For example, Thai society strongly emphasizes hierarchy and respect for authority, which is reflected in student-faculty relationships that can lead to unique stressors. Social media also plays a significant role in the lives of Thai students. These factors should be considered when designing future surveys to ensure that context-specific stress and burnout indicators relevant to Thai students are measured. Assessing the influence of social media and local culture on students’ burnout and stress levels should also be studied.

Furthermore, future research should explore the underlying factors that contribute to elevated burnout levels among Thai university students. These factors could include academic pressure, family expectations, or societal norms regarding academic success and performance. Understanding theses contributors could facilitate the development of tailored interventions and programs designed to prevent or reduce burnout in this population. In addition, in future research studies, we propose conducting a qualitative phase, using open-ended questions or focus group discussions, to collect data on potential causes of burnout and psychological stress in university life, as well as conducting in-depth interviews with students to assess their perceptions of burnout and indicators reflecting such symptoms.

## Conclusion

This study demonstrates that the MBI-SS and its components have high reliability, validity, and strong intercorrelations, highlighting the efficacy of the MBI-SS-International in assessing burnout among Thai university students. The findings suggest that the MBI-SS-Thai version is a suitable tool for evaluating burnout, particularly for identifying academic discouragement, emotional stress, or feelings of disengagement. Additionally, the instrument proves useful for guiding psychological counselling efforts and preventing adverse outcomes such as student dropout, absenteeism, or issues related to mental and physical health. These results are valuable for university administrators in developing strategies to enhance students’ adaptability, resilience, and overall well-being, ultimately facilitating their transition into both academic and professional environments.

## Supplementary Information

Supplementary Information.

## Data Availability

Data is provided within the supplementary information file. Permission to use the MBI questionnaire was obtained under a licensed agreement from Mind Garden, Inc.
